# Precise nanoscale temperature mapping in operational microelectronic devices by use of a phase change material

**DOI:** 10.1038/s41598-020-77021-1

**Published:** 2020-11-18

**Authors:** Qilong Cheng, Sukumar Rajauria, Erhard Schreck, Robert Smith, Na Wang, Jim Reiner, Qing Dai, David Bogy

**Affiliations:** 1 Department of Mechanical Engineering, UC Berkeley, California 94720 USA; 2 Western Digital Corporation, Recording Sub System Staging and Research, San Jose, CA 95135 USA

**Keywords:** Engineering, Nanoscience and technology, Applied physics, Condensed-matter physics, Electronics, photonics and device physics, Techniques and instrumentation

## Abstract

The microelectronics industry is pushing the fundamental limit on the physical size of individual elements to produce faster and more powerful integrated chips. These chips have nanoscale features that dissipate power resulting in nanoscale hotspots leading to device failures. To understand the reliability impact of the hotspots, the device needs to be tested under the actual operating conditions. Therefore, the development of high-resolution thermometry techniques is required to understand the heat dissipation processes during the device operation. Recently, several thermometry techniques have been proposed, such as radiation thermometry, thermocouple based contact thermometry, scanning thermal microscopy, scanning transmission electron microscopy and transition based threshold thermometers. However, most of these techniques have limitations including the need for extensive calibration, perturbation of the actual device temperature, low throughput, and the use of ultra-high vacuum. Here, we present a facile technique, which uses a thin film contact thermometer based on the phase change material $Ge_2 Sb_2 Te_5$, to precisely map thermal contours from the nanoscale to the microscale. $Ge_2 Sb_2 Te_5$ undergoes a crystalline transition at $\hbox {T}_{{g}}$ with large changes in its electric conductivity, optical reflectivity and density. Using this approach, we map the surface temperature of a nanowire and an embedded micro-heater on the same chip where the scales of the temperature contours differ by three orders of magnitude. The spatial resolution can be as high as 20 nanometers thanks to the continuous nature of the thin film.

The fundamental understanding of thermal dissipation in an integrated chip^1–5^ requires the development of a versatile technique capable of reliably mapping the areal temperature of various components integrated in the chip ranging from nanometer to micrometer dimensions^6,7^. Various thermometers were developed to achieve this goal^6,8–21^ and can be broadly classified into two categories: non-contact and contact based thermometers. Techniques such as Raman^9^, fluorescence^22^, luminescence^23^ and transmission electron microscopy^13,24,25^ are non-contact thermometers. However, the areal resolutions of these methods are limited either by the optical diffraction limit^26^ or by the use of specific metals and semiconductors^13^. A scanning thermal microscope is an extensively used contact thermometer, but it typically suffers from contact-related artifacts that lead to an under prediction of the device temperature. This is due to the thermal coupling strength between the device and the SThM tip, which is material dependent and difficult to measure^18,27^.

**Figure 1:**
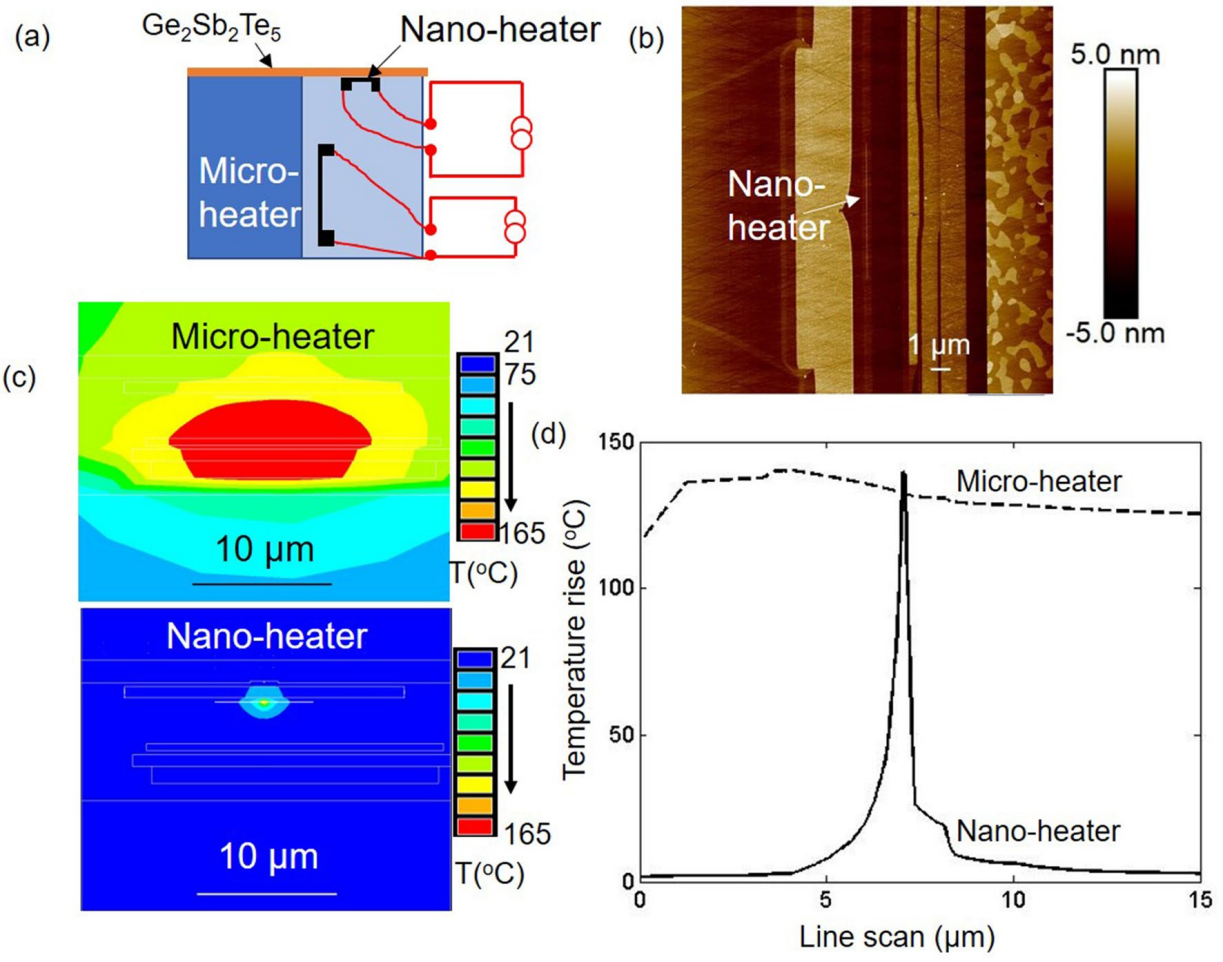
Heat sources inside the head of the hard disk drive. (**a**) Cross-sectional cartoon of the head structure showing the embedded heat sources: the nano-heater and the micro-heater. (**b**) AFM image of the device: the micro-heater is embedded and cannot be seen from the surface, while the nano-heater is located at the center. The dimension of the nano-heater is 1 $\upmu$m $\times$ 20 nm. (**c**,**d**) Simulation: temperature map of the nano-heater and the micro-heater with similar peak surface temperature.

Here a novel technique, which uses a phase change material to map the temperature of an operational microelectronic device, is presented. It requires minimal effort in temperature calibration and the temperature contour can be mapped using both contact and non-contact modes such as AFM, SEM or optical microscopes^28–32^. We map the temperature contours of a nanowire and an embedded micro-heater where the contour areas differ by three orders of magnitude.

To demonstrate the versatility and practicality of this technique, a recording head from a commercial hard disk drive is used. The head of the hard disk drive provides a unique platform for such studies as it has several embedded heat sources, which differ in heated area by three orders of magnitude^33,34^. At the microscale, it has a micro-heater, which is used to adjust the clearance between the head and the rotating disk^35^. The micro-heater is embedded a few micrometers from the surface, and it produces a microscale temperature contour. At the nanoscale, it has a nano-heater, which is used both as a heater and a thermometer. The nano-heater consists of a 200 nm wide, 1 $\upmu$m long, and 20 nm thick metal wire that is embedded 2 nm from the surface. Figure 1c shows the simulated surface temperature contours for the micro-heater and the nano-heater. In comparison to the micro-heater’s temperature field, the nano-heater’s areal temperature map is three orders of magnitude smaller (see Fig. 1d). Note that the nano-heater has a temperature coefficient of resistance (TCR) of 0.003/K, which is used to measure the average surface temperature. In this paper, it is quantitatively demonstrated that the temperature measured from the phase change temperature contour (PCTC) technique agrees well with the measured average surface temperature and the thermal simulation for both the micro-heater and the nano-heater.

## Results and discussions

### Self-heating of the nano-heater

**Figure 2:**
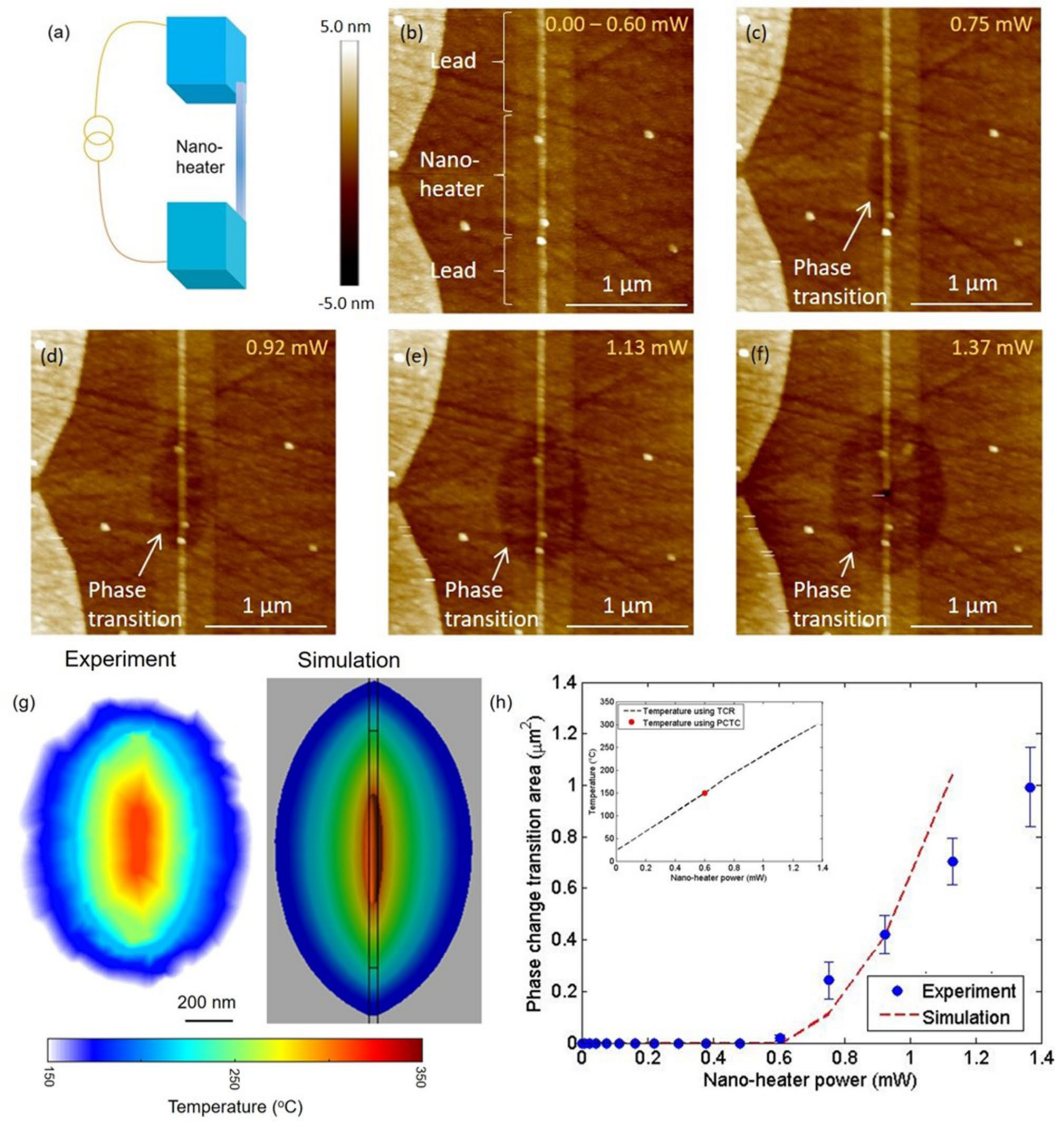
Self-heating of the nano-heater. (**a**) Schematic diagram of the nano-heater. Nanowire with dimension 1 $\upmu$m $\times$ 20 nm $\times$ 200 nm is electrically connected to two pads. (**b**–**f**) The AFM images of the device at different micro-heater bias conditions. (**c**–**f**) The depression in the topography from the phase transition around the nano-heater. (**g**) The constructed temperature contour from the PCTC technique and the simulation for the nano-heater power of 1.37 mW. (**h**) The measured phase change transition area as a function of dissipation power in the nano-heater power. The red dash line corresponds to the simulation of an isotherm contour for the glass transition temperature $\hbox {T}_g$. In the inset, the black dash line shows the estimated average surface temperature along the nano-heater from the resistance change in the nano-heater and the measured isotherm from the PCTC technique (red dot). Estimated error bar in average surface measurement is 0.04 $^{\circ }$C.

**Figure 3:**
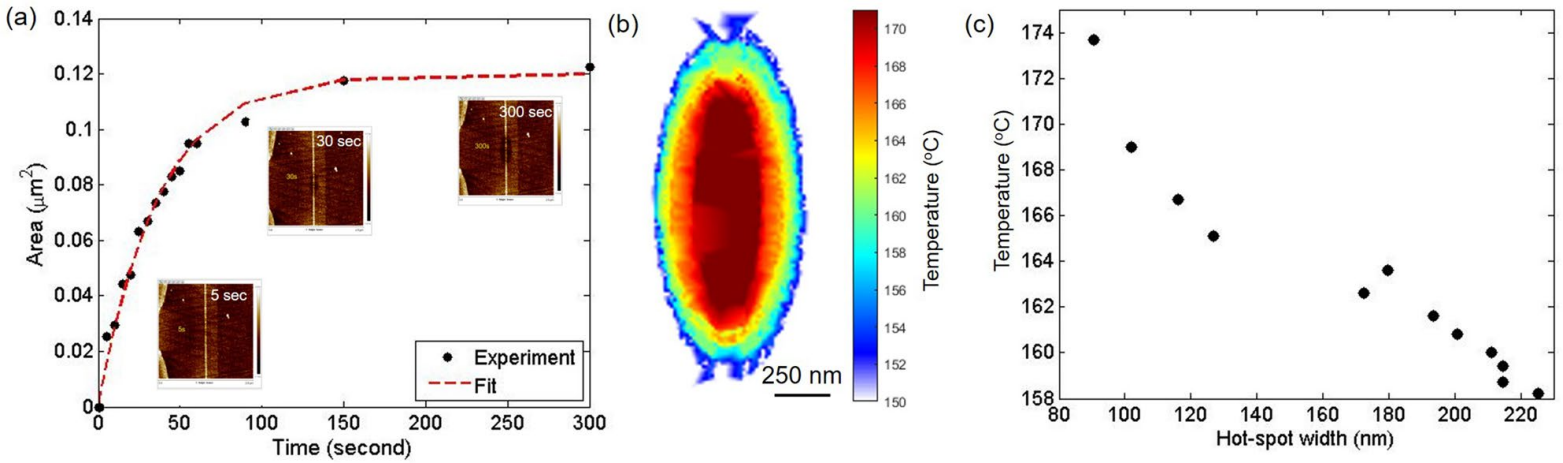
Time response of $Ge_2 Sb_2 Te_5$ at a constant nano-heater power of 0.68 mW. (**a**) The transition area of Ge$_2$ Sb$_2$ Te$_5$ phase change with the the accumulated heating time. (**b**) The temperature map of the nano-heater. (**c**) The temperature as a function of the hotspot width across the nano-heater.

To demonstrate the technique, we first characterize the Joule heating of the nano-heater inside the head of the hard disk drives. The head surface is coated with a 22 nm thick layer of $Ge_2 Sb_2 Te_5$ thin film. The nano-heater is biased using a current source, across which the measured voltage drop is used to estimate the resistance increase of the nano-heater due to the dissipated Joule heat (Fig. 2a). The resistance change of the nano-heater is used to estimate the average temperature increase of the device temperature by $R_{T}=R_{0}(1+\alpha \Delta T)$, where $R_{0}$ is the room-temperature resistance at low current bias where no significant self-heating occurs, $R_{T}$ is the resistance at the bias corresponding to the temperature T, $\alpha$ is the temperature coefficient of resistance (TCR), and $\Delta$T is the average temperature rise due to the Joule heating. The temperature coefficient of resistance ($\alpha$) 0.003/K is determined separately in an oven using a 4-probe measurement scheme (see Supplementary section 1). Note that the effect of the thin layer on the heat transport of the system is negligible (see Supplementary section 2).

The amorphous $Ge_2 Sb_2 Te_5$ is a chalcogenide phase change material that crystallizes at $\hbox {T}_{g}\sim 149^{\circ }$C for a dwell time of 5 min. This crystallization is accompanied by an increase in density and volume reduction, where AFM topography measurement shows as a reduction in the film height. Figure 2b–f show the AFM topography micrographs corresponding to different powers in the nano-heater. For nano-heater power smaller than 0.60 mW, the AFM shows no change in the topography of the $Ge_2 Sb_2 Te_5$ film over the nano-heater, indicating that the surface temperature is lower than the crystallization temperature. When the nano-heater power is 0.75 mW, a small depression in the topography is observed centered at the hot spot of the nano-heater as shown in Fig. 2c. A further increase in the nano-heater power leads to a gradual increase in the area undergoing the crystallization, which is indicated by the lateral growth of the depressed area in the AFM images. Note that the boundary of the topography depression corresponds to the isotherm of the crystallization temperature. The evolution of the temperature contour area agrees reasonably well with the simulation as shown in Fig. 2h. Furthermore, we use the transition boundary measured at different nano-heater powers to map the temperature of the device. The rate of phase transition in $Ge_2 Sb_2 Te_5$ is a function of both the temperature and the time. Here, the power in the nano-heater is increased incrementally with a fixed dwell time until the initial transition boundary is observed. The last transition boundary corresponds to the calibration temperature $\hbox {T}_{{g}}$ at the largest heater power $\hbox {P}_{{o}}$=1.37 mW (Fig. 2f). Assuming that the temperature is linear with the applied power, the temperature isotherm $\hbox {T}_{{i}}$ at each previous transition boundary (Fig. 2c–e) is given by:

1 $$\begin{aligned} T_{i}=T_{g}\frac{P_{o}}{P_{i}} \end{aligned}$$

where $\hbox {T}_{{g}}$ is the calibrated $Ge_2 Sb_2 Te_5$ crystalline transition temperature for the dwell time of 300 s during which the nano-heater is powered on, $\hbox {P}_{{o}}$ is the nano-heater power at which the final transition boundary is measured, and $\hbox {P}_{{i}}$ is the previous power with $\hbox {P}_{{i}}$ < $\hbox {P}_{{o}}$ in the nano-heater. Figure 2g shows the constructed temperature map of the device along with the simulation for $\hbox {P}_{{o}}$ = 1.37 mW.

**Figure 4:**
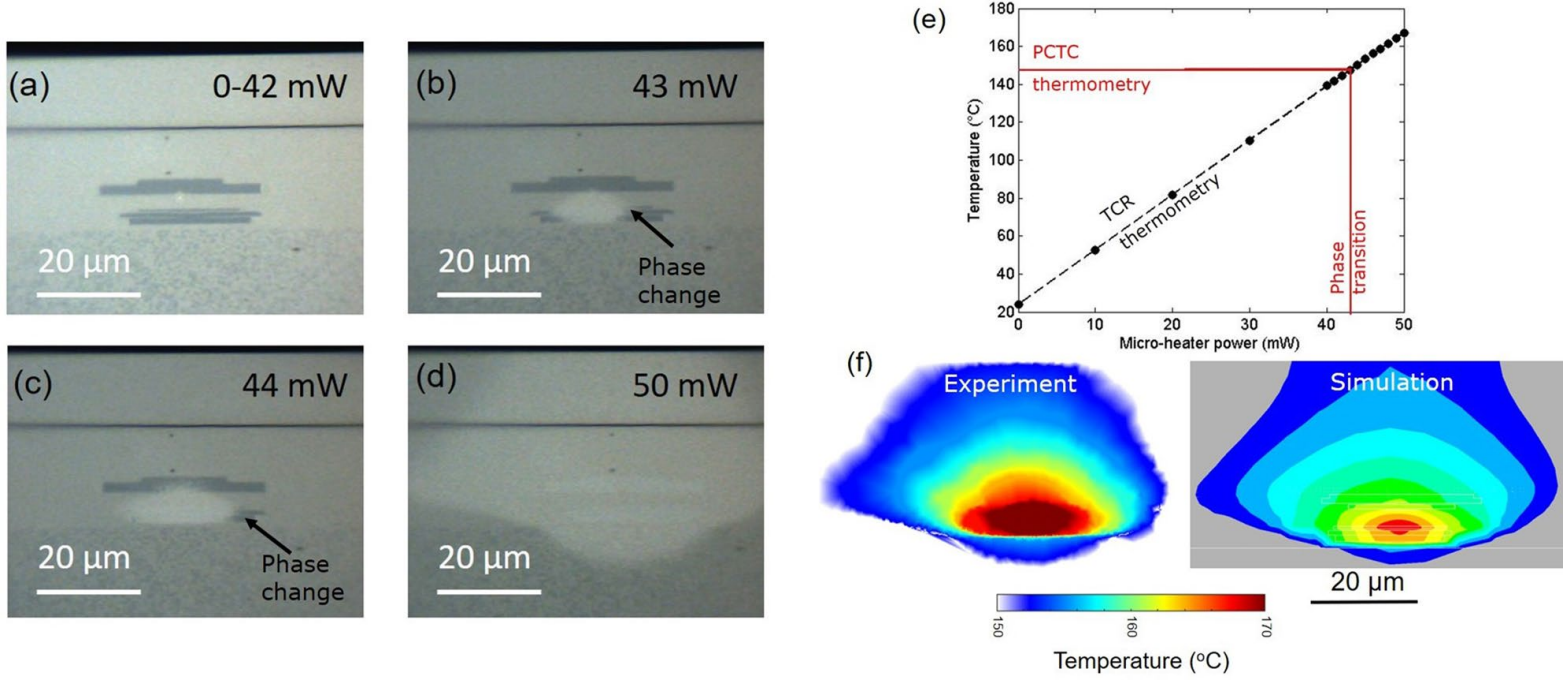
Self-heating of the micro-heater. (**a**–**d**) Optical micrographs of the device at different micro-heater powers. (**b**–**d**) The reflectivity increase at the center of the micrographs corresponding to the phase change due to the temperature rise of the micro-heater. (**e**) Shows the average surface temperature measured by the nano-heater, acting as a thermometer, along with the critical point for which the phase transition is measured from the optical micrograph. Estimated error bar in average surface measurement is 0.04 $^{\circ }$C. (**f**) The constructed temperature map of the device along with the simulation at the micro-heater power of 50 mW.

Figure 2h shows the phase change transition area calculated from the topography depression in the AFM image as a function of the power dissipated in the nano-heater. When the nano-heater power is lower than 0.60 mW, the transition area is zero signifying that the surface temperature is lower than the glass transition temperature $\hbox {T}_{{g}}$ everywhere. At higher powers the transition area grows linearly with the dissipated power in the nano-heater. The simulation results are shown as the red dash line. Both the experiment and the simulation show a sharp increase in the phase change transition area beyond 0.60 mW. At much larger bias currents the poor match is because of our inability to capture the exact structural details in the simulation such as the actual thermal boundary conditions and the various material parameters. To further confirm the surface temperature, we simultaneously measure the resistance of the nano-heater and use a TCR of 0.003/K to estimate the average temperature of the heater. Figure 2h inset shows the measured surface temperature of the nano-heater as a function of the power dissipated. The red dot is the temperature from the PCTC technique at 0.60 mW derived from the x-axis intercept of the Fig. 2h. As expected, the temperature from the PCTC technique matches the temperature reading given by the resistance change. The excellent agreement between the temperature measured using the PCTC and TCR technique along with the simulation demonstrates that the PCTC technique can precisely map the high operational temperature of the nanoscale heater embedded in the chip.

Next we study the time response of the $Ge_2 Sb_2 Te_5$ film to construct the temperature map of the device at a constant nano-heater power. Here, the growth of the transition boundary is tracked over time. It should be noted that the transient response of the nano-heater is six orders of magnitude faster than the $Ge_2 Sb_2 Te_5$ phase change. Assuming that the phase change conversion follows an Arrhenius model and the conversion is linear with time, the temperature for each transition boundary is derived by monitoring the time needed for each transition boundary to develop. The temperature ($T_{i}$) at time $t_{i}$ is given by:

2 $$\begin{aligned} T_{i}(t_{i})=\left[ -\frac{k_{B}}{E_{A}}\left( ln\frac{1}{t_{i}} - ln\frac{1}{t_{cal}} \right) + \frac{1}{T_{g}(t_{cal})} \right] ^{-1} \end{aligned}$$

where $k_{B}$ is the Boltzmann constant, $E_{A}$ $\sim$ 2.6 eV is the activation energy $Ge_2 Sb_2 Te_5$ transition, $\hbox {T}_{g} \sim$ 149 $^{\circ }$C is the calibrated crystallization temperature at dwell time $\hbox {t}_{{cal}}$ =300 s. It is worth noting that the temperatures of isotherms corresponding to shorter dwell times ($t_{i}$ < $t_{cal}$) are higher than $\hbox {T}_{{g}}$.

Figure 3a dots show the phase change transition area around the nano-heater as a function of the accumulated time for a constant power of 0.68 mW (see Supplementary section 3 and Video S3). The red line is the exponential fit with a time constant of 37.6 s. The transition temperature at different accumulated heating time is determined by using Eq. (2). Figure 3b shows the constructed temperature map of the device at the constant nano-heater power of 0.68 mW. In comparison to Fig. 2g, the temperature map is smaller and more elliptical since the nano-heater power is almost 50$\%$ smaller. Figure 3c shows the temperature across nano-heater as a function of distance demonstrating the high resolution of the PCTC scheme. The continuous nature of our thin film allows for a higher spatial resolution, which is limited only by the grain size of $Ge_2 Sb_2 Te_5$ (sub 20 nm) and the resolution of the imaging technique (< 10 nm)^14^.

### Self-heating of the micro-heater

To demonstrate the versatility of the PCTC technique, we now characterize the Joule heating of a much larger micro-heater embedded in the head of the hard disk drive. The temperature contour of the micro-heater is three orders of magnitude larger than that of the nano-heater embedded in the same chip (as shown in Fig. 1) in terms of the contour area. The micro-heater is biased using a current source and the measured voltage drop across the nano-heater (thermometer) is used to estimate the dissipated Joule heat. The dwell time of 300 s at a constant micro-heater power is much longer than the thermal response time of the heater and the phase transition time beyond which the physical, optical and electrical properties change. The temperature contour of the micro-heater is mapped using an optical microscope by simply imaging the reflectivity change in the transition area. Figure 4a–d show the optical micrographs at different micro-heater powers. No change in reflectivity is observed below the dissipated power of 42 mW in the micro-heater. At the power of 43 mW, an increase in the reflectivity is observed at the center of the thermal hotspot due to the micro-heater. Note that the boundary of the transition area corresponds to the crystallization temperature. In comparison to the nano-heater, here the micro-heater requires 60 times more power to achieve the same surface temperature since the micro-heater is more deeply embedded and heats up a much larger volume. At higher micro-heater powers, the growth in the transition area indicates an increase in the thermal spot size with the same crystallization temperature $\hbox {T}_{{g}}$ isotherm.

Figure 4f shows the constructed temperature map of the device along with the simulation. The dimensions and the overall shape of the transition contour from the experiments match well with the simulated $\hbox {T}_{{g}}$ isotherms. Furthermore, we utilized the nano-heater as a thermometer by monitoring its resistance change at a very low current of 0.1 mA, in order to avoid self-heating, to measure the temperature rise due to the micro-heater. Figure 4e shows the surface temperature measured by the nano-heater as a function of the power dissipated in the micro-heater. The red line shows the micro-heater power beyond which the phase transition is observed in the optical micrograph. The expected rise of the surface temperature as derived from both the PCTC technique and from the nano-heater (acting as ‘thermometer’) is 2.9 K/mW. This shows an excellent agreement between the PCTC technique, the measured surface temperature and the simulation for the temperature map of the micro-heater.

**Figure 5:**
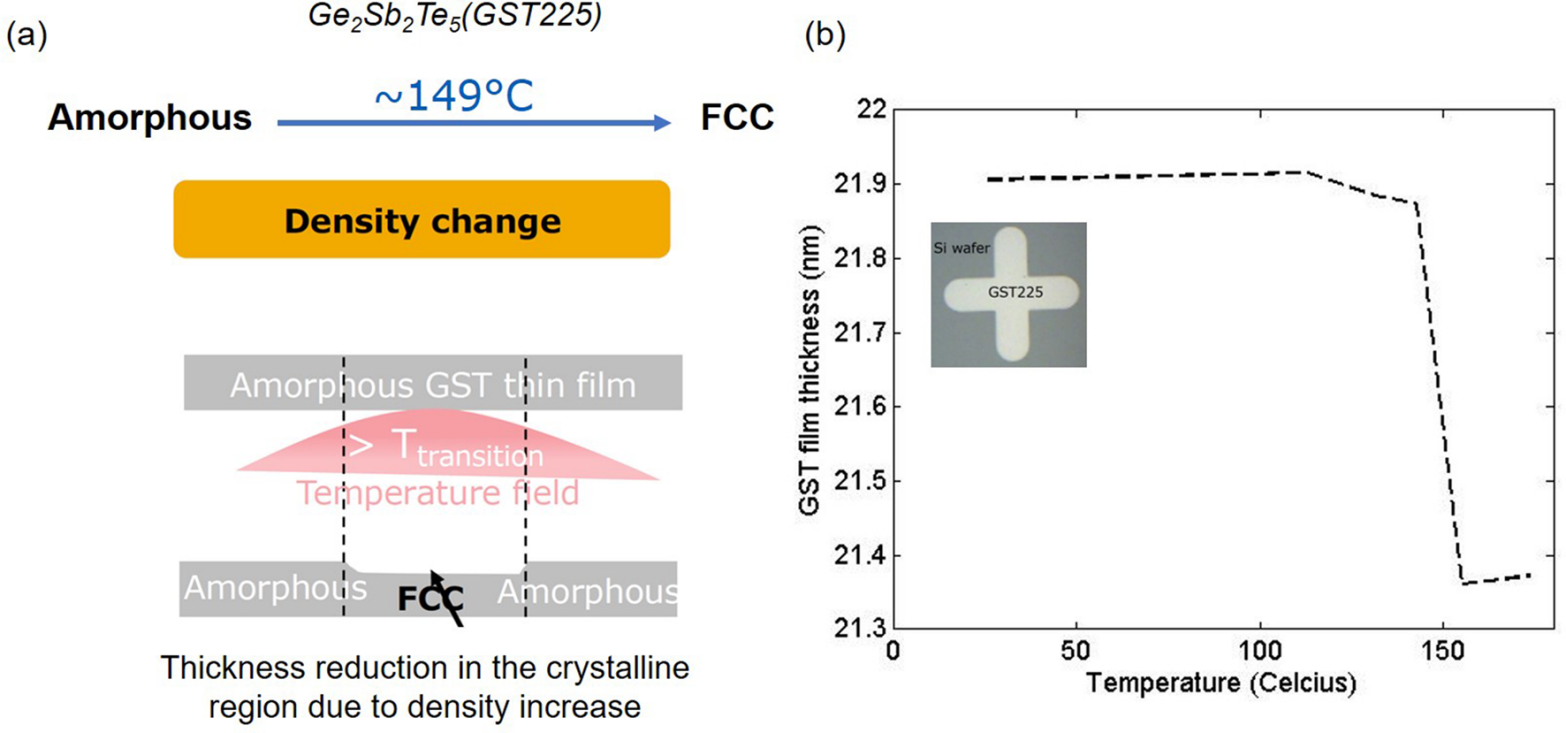
Calibration. (**a**) Cartoon to show the phase transition in $Ge_2 Sb_2 Te_5$. (**b**) Inset: optical micrograph of the calibration sample. Main: thickness (measured using AFM, with vertical resolution of 0.05 nm) of the calibration sample as a function of oven temperature. The glass transition temperature $\hbox {T}_{{g}}$ is 149 $^{\circ }$C.

### Temperature calibration

Precise and relatively simple temperature calibration is a key advantage of the PCTC method compared to other techniques that require extensive temperature calibration. To calibrate the crystallization temperature $\hbox {T}_{{g}}$ of the phase change material $Ge_2 Sb_2 Te_5$, we rely on a pronounced structural property change at the phase change condition (Fig. 5a). The rate of this transformation from an amorphous state to the crystalline rock salt structure is well characterized by an activation energy of about 2.6 eV^29,30^. Due to this activation energy driven process the crystallization temperature is dependent on the dwell time of the sample. For this reason, the dwell time at a constant power condition is fixed at 5 min for both the nano-heater and the micro-heater. This dwell time is much longer than the response time of the two heaters^36^. Figure 5b inset shows the calibration sample, which is a photo-lithography defined 22 nm thick $Ge_2 Sb_2 Te_5$ layer on top of a silicon wafer. The main figure shows the film thickness as a function of oven temperature. The dwell time at a constant oven temperature is 5 min after which the sample is allowed to cool down to room temperature, and the film thickness is measured using the AFM. At T = 149 $^{\circ }$C, the film thickness reduces, indicating a phase transition from the amorphous state to the crystalline state, as confirmed by the X-ray diffraction pattern (see Supplementary section 4).

The uncertainty in the temperature derived from this PCTC technique is primarily due to the fact that the crystallization rate of the phase change material does not have a large abrupt jump at a single temperature (as in a first order phase transition). As a result, the full temperature history of the sample, not just the last power used, can influence the size of the observed contour (see Supplementary section 5). For both the nano-heater and the micro-heater, the estimated temperature step is kept at 10 K, which leads to a slight under-prediction of the temperature by around 2 K at the 149 $^{\circ }$C crystallization condition.

It is noteworthy that in most other high resolution temperature mapping techniques, the application is limited to the large scale devices. Here we demonstrated the thermal measurements of the two heaters that produced thermal contours with dimensions that differ by three orders of magnitude, while maintaining sub 20 nm resolution in both cases. This technique is extremely versatile and does not require the use of expensive microscopes like STEM. At the microscale, even an inexpensive optical microscope can be used to map the temperature of the hotspots or heat sources in operating microelectronics devices.

Finally, the limitations of the presented PCTC technique are discussed. Although the technique is versatile and can be used for nanoscale to microscale spatial heat sources with minimal calibration challenges, it has two main limitations. First, the areal contour represents the isotherm at $\hbox {T}_{{g}}$, and to extract the temperature gradient one needs to perform mapping for at least two different power levels or track the phase change with time. Secondly, the technique is limited to a device temperature higher than the crystallization temperature of the deposited phase change material. In our case, $Ge_2 Sb_2 Te_5$ crystallizes at temperature of 149 $^{\circ }$C for the dwell time of 5 min is used. Both embedded heaters are able to reach temperatures higher than this $\hbox {T}_{{g}}$. For other systems where reaching a similar temperature would be difficult or impossible, the issue could be overcome by choosing a different composition or phase change materials with lower $\hbox {T}_{{g}}$^37–40^.

## Conclusion

To summarize, we introduce a versatile phase-change-material-based temperature mapping technique for operational microelectronic devices that can spatially resolve temperature from nanoscale to microscale dimensions. It can be used to characterize surface temperatures with neglectable temperature interference due to the deposited measurement film and with minimal calibration. A thorough understanding of the heat dissipation in various nanoscale devices, such as the aforementioned nano-heater, may lead to more efficient and powerful integrated chips, and hence holds great economic value to the industry.

## Methods

### Experimental set-up

The microelectronic device is held on a metal fixture with electrical pins. The components inside the device such as the heaters are powered by Keithley 2602 SYSTEM SourceMeter, which is controlled by a Python script. A 22 nm thin film of Ge2Sb2Te5 is sputtered on the surface of the device. The topography change or the reflectivity change of the thin film due to the heaters are measured by Digital Instruments Dimension 3100 AFM or an optical microscope respectively. In the temperature calibration of the Ge2Sb2Te5, a silicon wafer with a photo-lithography defined 22 nm thick Ge2Sb2Te5 layer is heated in a customized copper chamber, where the temperature is measured by a type-K thermocouple. The thickness of the layer is also measured by the AFM.

### Simulation

Thermal simulations of the micro-heater and nano-heater devices in Fig. 1c,d were performed using finite element models in ANSYS Mechanical APDL version 17.2. The nano-heater thermal simulation in Fig. 2g was modeled using a finite element model in the ANSYS Workbench Thermo-electric module version 17.2. The device surfaces were modeled using a convection cooling boundary condition with a coefficient of 50 W/($\hbox {m}^{2}$ K).

## Supplementary information

Supplementary Information.

Supplementary Video S1.

Supplementary Video S2.

Supplementary Video S3.
