# Supplementary material for: Precise nanoscale temperature mapping in operational microelectronic devices by use of a phase change material
Source: Sci Rep. 2020 Nov 18;10:20087. doi: 10.1038/s41598-020-77021-1 (PMC7674486; doi:10.1038/s41598-020-77021-1)
Supplement: Supplementary file 1 — Supplementary Information. [file 41598_2020_77021_MOESM1_ESM.pdf]

Precise nanoscale temperature mapping in operational microelectronic devices by use of a phase change material

Supplementary Information

Qilong Cheng[1a], Sukumar Rajauria[2a+], Erhard Schreck[2], Robert Smith[2], Na Wang[2], Jim Reiner[2], Qing Dai[2], and David Bogy[1]

[1]Department of Mechanical Engineering, University of California at Berkeley, Berkeley, CA 94720 USA.
[2]Western Digital Corporation, Recording Sub System Staging and Research, San Jose, CA 95135 USA.
[+]sukumar.rajauria@wdc.com
[a]Equal contribution.

Contents

1 TCR measurement for the nanowire 2
2 Effect of $Ge_2Sb_2Te_5$ thin film on the heat transport in our system 3
3 Time dependent PCTC 4
4 In-situ characterization of the phase change 5
5 Accumulation behavior of the PCM $Ge_2Sb_2Te_5$ 6
6 The transition area of $Ge_2Sb_2Te_5$ phase change vs. the accumulated heating time 7

1 TCR measurement for the nanowire

To measure the TCR of the nanowire (acting as thermometer), we put the nanowires in an isothermal Cascade Tek Oven with temperature control. Four-probe measurement scheme is used to determine the resistance of the nanowire and a K-type thermocouple is used to monitor the temperture inside the oven as shown in Figure S1.

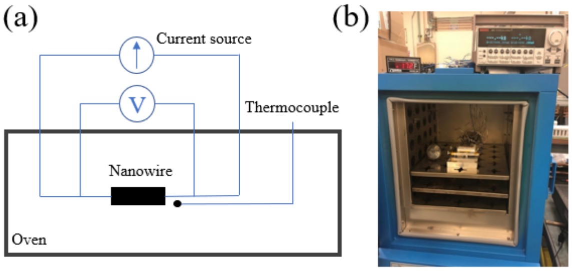

Figure S1. (a) Schematics of the experimental set-up. (b) The experimental set-up.

In the TCR measurement, the current source is at low bias of 0.1 mA such that the self-heating of the nanowire can be ignored (about 0.1 °C increase). The oven temperature rises from room temperature to around 115 °C in steps. For each temperature, the resistance and the reading of the thermocouple are recorded when steady. The slope in Figure S2 is exactly TCR α and all three samples show the same TCR 0.003/°C. Therefore, the nanowire, with known TCR, can be used as a measure of the temperature.

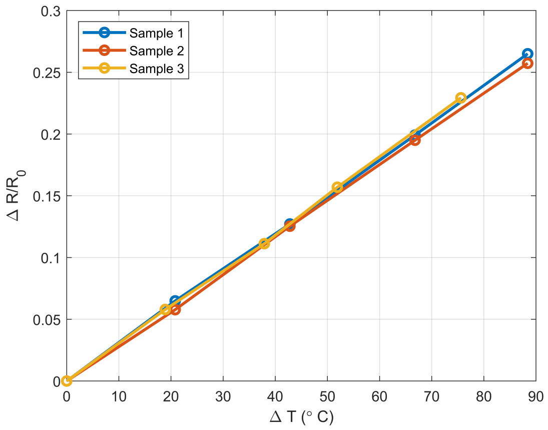

Figure S2. The $\Delta R/R_0$ as a function of ΔT for three samples.

2 Effect of $Ge_2Sb_2Te_5$ thin film on the heat transport in our system

In this section, we show that the effect of $Ge_2Sb_2Te_5$ thin film on the heat transport in our system is negligible. Figure S3 shows the measured average temperature of the nanowire as a function of its power before and after $Ge_2Sb_2Te_5$ coating. Both two samples show that the $Ge_2Sb_2Te_5$ coating has almost no effect on the thermal transport (~ 0.2 °C difference, which is negligible). Figure S4 shows the temperature profile across the nanowire (along the minor axis of the transition contour) from the simulations. In the center of the nanowire, the simulations show a 0.2 °C difference, which matches well with results shown in Figure S3. Considering that the dimension of the transition area due to nanowire self-heating (< 0.5 μm), the temperature difference caused by $Ge_2Sb_2Te_5$ thin film is ~ 0.2 °C, which is small and acceptable. It is worthwhile noting that an interface conductance of 100 MW/(m^2K) is considered between the device surface and $Ge_2Sb_2Te_5$ film in the simulations.

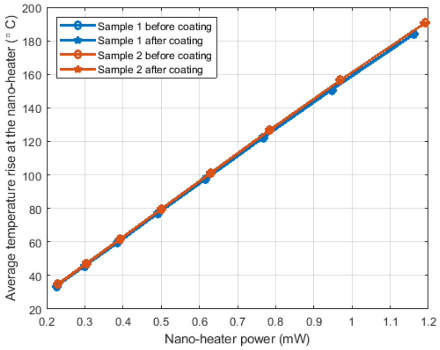

Figure S3. The average temperature as a function of the nano-heater power.

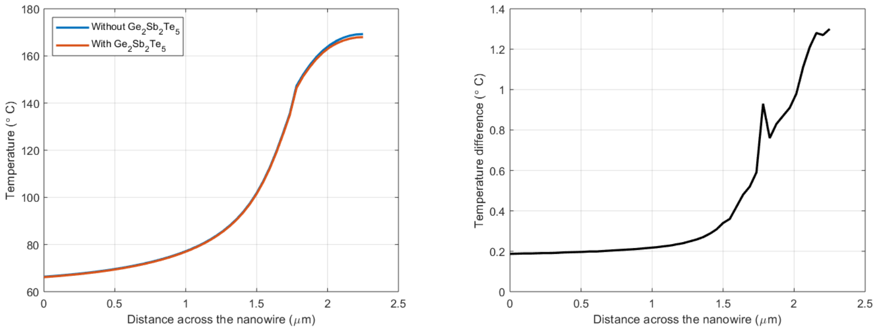

Figure S4. The simulated temperature profile across the nanowire with/without presence of $Ge_2Sb_2Te_5$ film

3 Time dependent PCTC

In our phase change temperature contour (PCTC) technique, the phase change material (PCM) $Ge_2Sb_2Te_5$ undergoes a phase change along with changes in electric conductivity, optical reflectivity and density. But the transition takes time to fulfill. In this section, we design a time dependent PCTC experiment to study the time it takes to complete transition.

In the time dependent PCTC experiment, the nano-heater is biased at a constant nano-heater power of 0.68 mW. $Ge_2Sb_2Te_5$ senses the self-heating of the nanowire and begins to transit from amorphous to FCC state. After a certain heating time, the nano-heater is turned off and the surface topography of the nanowire is characterized by AFM. Then repeat the step until the transition is complete. The attached video "Time_Dependence.mp4" shows a video of phase change area at the center of the nanowire from 0 to 300 s. The contour has a shape of perfect ellipse. The long and short axes are measured to calculate the area of the transition. Figure S5 shows the result of transition area with the accumulative time and the corresponding curve fitting using an exponential function. It is revealed that the time constant for such a nanowire self-heating scheme is 37.6 s and it takes 3.1 minutes for the transition to fulfill.

Obviously, 3.1 minutes is also sufficient for micro-heating scheme to attain the equilibrium because the heating has a broader area which is three orders of magnitude larger than the nanowire self-heating scheme. Subsequently, the dwell time for each heating experiment is always kept as 5 minutes such that the phase change of $Ge_2Sb_2Te_5$ is totally completed, namely the contour is in steady state.

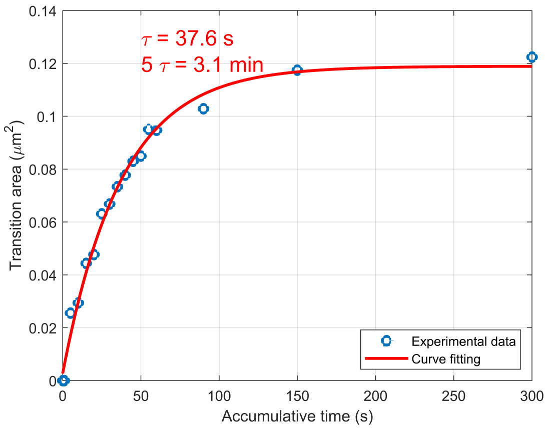

Figure S5. The transition area of $Ge_2Sb_2Te_5$ phase change with the accumulative time for the bias current 2.5 mA through the nanowire

4 In-situ characterization of the phase change

In this section, the kinetics of the phase change from the amorphous state to crystalline is further studied using transmission electron microscopy (TEM). A 22 nm film of $Ge_2Sb_2Te_5$ is deposited on a SiC substrate. The sample is heated in-situ such that the electron diffraction analysis and TEM imaging are performed in real time.

Figure S6 shows the result of the electron diffraction patterns and TEM images. Before the critical temperature $T_g = 149°C$, (a) shows that the material is amorphous. Then the sample is soaked at $T_g = 149°C$ for 1 min as (b), some diffraction spots appear indicating that the phase change begins. After soaking for 5 mins, several orientations of the crystalline form clearly. Meanwhile, (d) and (e) are the TEM images before/after the phase change. It is obvious that $Ge_2Sb_2Te_5$ undergoes the phase change from amorphous to crystalline.

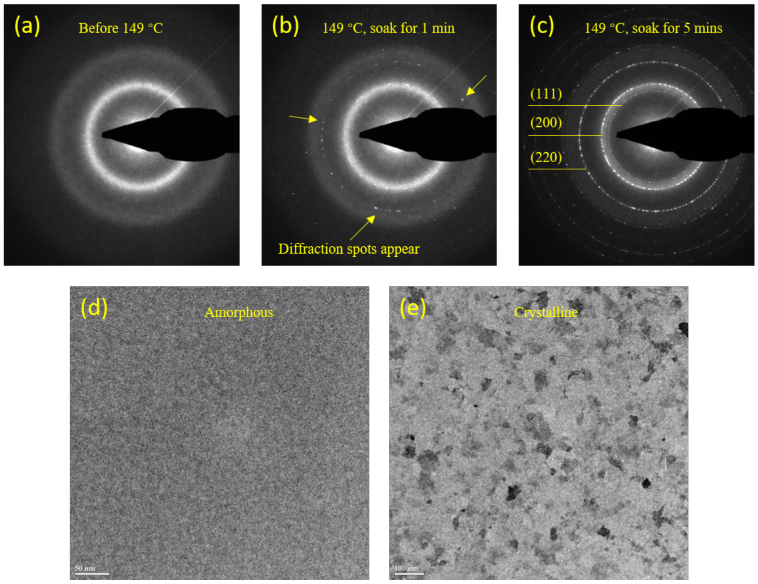

Figure S6. (a)-(c) Show the electron diffraction pattern of $Ge_2Sb_2Te_5$ before the critical temperature and soak at the critical temperature (1 min/5 mins). (d)(e) Show the TEM of $Ge_2Sb_2Te_5$ before and after the phase change.

5 Accumulation behavior of the PCM $Ge_2Sb_2Te_5$

The activation energy E_a to undergo a structural change from an amorphous to rock salt FCC structure is around 2.6 eV. When temperature T is below the glass transition temperature T_g, there is still a small proportion of the material that transits to FCC. The proportion can be expressed as

$$P_i = e^{-\frac{E_a}{k_B}(\frac{1}{T_i}-\frac{1}{T_g})} \tag{1}$$

where P_i refers to the proportion of transition at the temperature T_i and k_B is Boltzmann constant. Therefore, when we heat the same material in multiple heating cycles (N cycles), the total proportion of transition is the summation of all the previous heating cycles:

$$P = \sum_{i=1}^{i=N} P_i \tag{2}$$

Figure S7 plots the modelling result of the transition proportion of $Ge_2Sb_2Te_5$ with temperature for single heating and accumulating heating. In the single heating, 100% of the material transits at 149 °C. However, as for accumulating heating, all the heating cycles have contribution to the phase change although below the critical temperature T_g, so finally the measured critical temperature corresponded to 100% transition is about 2 °C below the real value, which is acceptable.

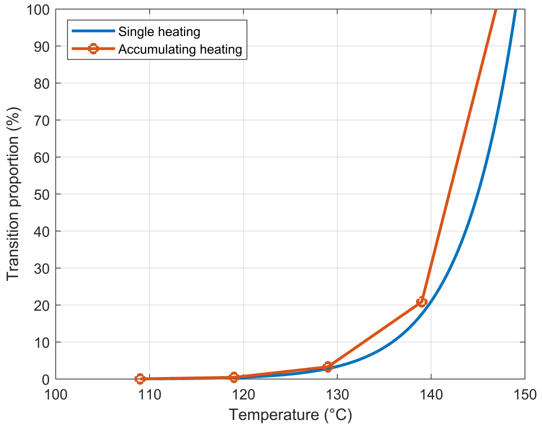

Figure S7. The transition proportion of the PCM with temperature for single heating and accumulating heating

In the following experiments of the nano-heater self-heating or micro-heater heating schemes, the temperature step is maintained above 10 °C such that the accumulation behavior of the phase change becomes negligible.

6 The transition area of $Ge_2Sb_2Te_5$ phase change vs. the accumulated heating time

Figure S8 shows the bigger sized version of Fig. 3(a) inset. The AFM images of the transition area vs. the accumulated heating time are shown.

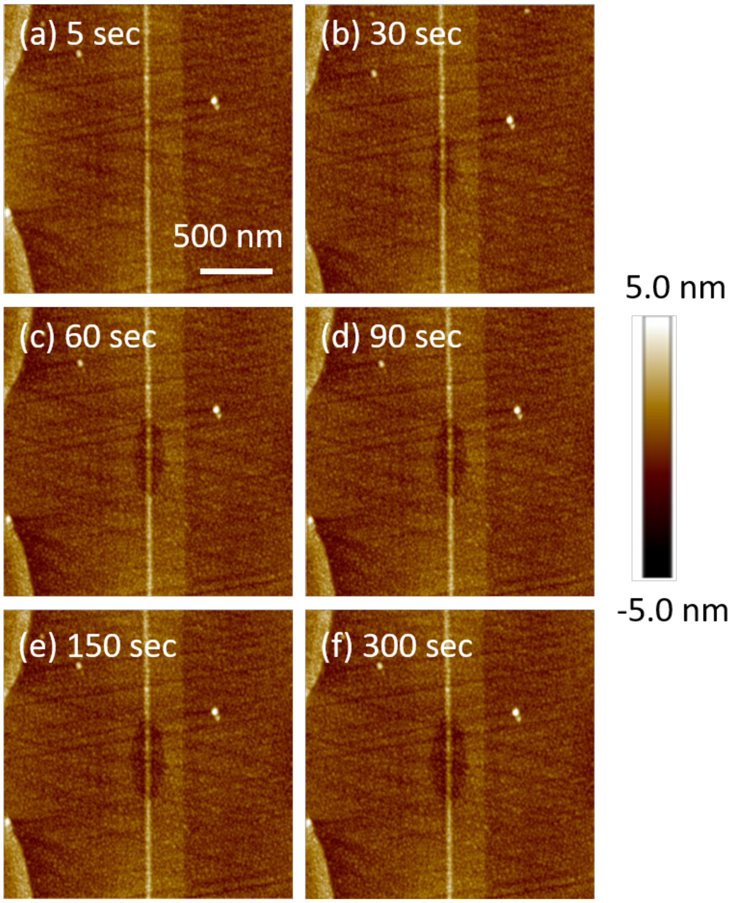

Figure S8. The transition area of $Ge_2Sb_2Te_5$ phase change vs. the accumulated heating time at a constant nano-heater power of 0.68 mW
